# High-resolution structural connectivity mediates age-related differences in functional connectivity and fluid cognition

**DOI:** 10.1093/braincomms/fcaf376

**Published:** 2025-09-28

**Authors:** Jenna L Merenstein, Allen W Song, David J Madden

**Affiliations:** Department of Psychology, University of Utah, Salt Lake City, UT 84112, USA; Brain Imaging and Analysis Center, Duke University Medical Center, Durham, NC 27710, USA; Brain Imaging and Analysis Center, Duke University Medical Center, Durham, NC 27710, USA; Department of Radiology, Duke University Medical Center, Durham, NC 27710, USA; Brain Imaging and Analysis Center, Duke University Medical Center, Durham, NC 27710, USA; Department of Psychiatry and Behavioral Sciences, Duke University Medical Center, Durham, NC 27710, USA

**Keywords:** graph theoretical analyses, multiplexed sensitivity encoding, diffusion-weighted imaging, healthy aging, white matter

## Abstract

Magnetic resonance imaging studies using diffusion-weighted imaging suggest that age-related cognitive decline and alterations in brain function, in healthy adults, are at least partly explained by the degradation of white matter pathways connecting distributed brain regions. Studies of younger adults and animal models suggest that more precise estimates of white matter connectivity may be achieved by higher resolution, relative to standard spatial resolution, diffusion-weighted imaging. Here, in a cross-sectional study of healthy adults across the lifespan (*n* = 140; ages 18–88 years; 72 females), we compared age-related differences in measures of white matter structural connectivity from standard (1.5 mm^3^ voxels; 3.375 µl volume) and high-resolution (1 mm^3^; 1 µl volume) diffusion-weighted imaging, and their ability to explain age-related differences in functional connectivity and cognition. We assessed cognition using tests of memory, executive function, and perceptual-motor speed, and assessed structural and functional (resting-state functional magnetic resonance imaging) connectivity using graph theory. Results revealed more pronounced age-related decreases in structural connectivity for sensorimotor, ventral attention, and sub-cortical networks for high-resolution than standard diffusion-weighted imaging. Age-related decreases in functional connectivity were evident across the brain and mediated by high-resolution structural connectivity in the default mode network. Age-related decline in fluid cognition was mediated by within-network connectivity from only high-resolution diffusion-weighted imaging, but by a combination of high-resolution and standard diffusion-weighted imaging for between-network connectivity. Thus, relative to standard diffusion-weighted imaging, high-resolution diffusion-weighted imaging may better capture age-related differences in white matter connectivity and their constraint on age-related alterations in brain function and cognitive performance.

## Teaser

Merenstein *et al*. reported that structural brain connectivity, as measured by high-resolution diffusion-weighted MRI, improved the ability to predict age-related alterations in brain function and cognitive performance, relative to standard spatial resolution. These results may assist with the early identification of adults who are at heightened risk for cognitive decline.

## Graphical Abstract

**Graphical Abstract:**
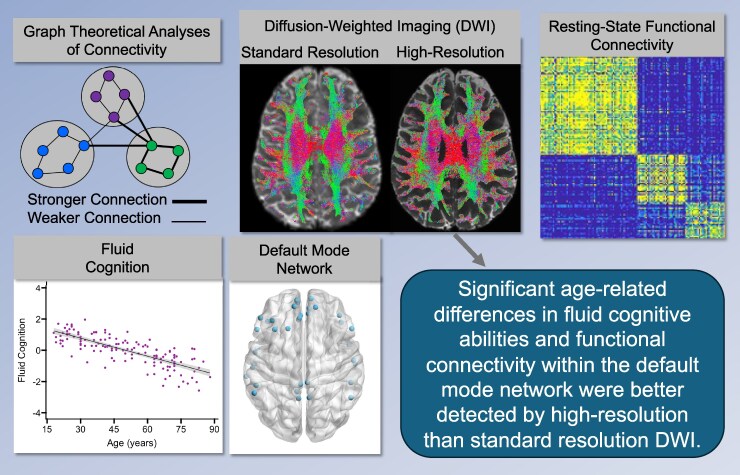

## Introduction

Healthy aging is accompanied by some degree of decline in fluid, speed-dependent cognition.^1-5^ Theories based on studies assessing brain structure using MRI techniques such as diffusion-weighted imaging (DWI) propose that the disconnection of white matter (WM) pathways between distributed brain regions contributes to age-related cognitive decline.^6-9^ However, higher resolution DWI acquisitions may help us better understand age-related structural disconnection, and its relation to brain function, which is necessary to inform future interventions aimed at slowing neurocognitive aging.

White matter plays a crucial role in cognitive function, such that heavily myelinated fibres propagate action potentials among different grey matter (GM) regions in an efficient manner.^7,10,11^ In theory, declines in DWI measures of WM structural connectivity should constrain functional MRI (fMRI) measures of GM functional connectivity in healthy aging,^12,13^ such that faster axonal propagation of action potentials between pre- and post-synaptic neurons should benefit cognitive performance.^7^ However, studies report mixed results regarding the presence and direction of these structure-function associations in adults across the lifespan.^14-17^ For example, some studies have identified significant age-related decreases in the association between brain structure and brain function,^18,19^ and other studies have reported relatively independent age-related effects on structural and functional connectivity.^16,17,20,21^ One possibility for this discrepancy in the literature is that functional connectivity measurements vary with task demands (e.g. cognitive load versus resting-state), whereas WM connectivity is instead relatively static at the time of measurement.^22,23^ A second possibility, to be explored here, is that these mixed findings may be due to the relatively low spatial resolution of standard DWI acquisitions attenuating the ability to detect these associations. Compared to standard DWI, higher resolution DWI should better estimate WM fibres in small, tightly curved regions, and voxels with multiple tissue types,^24-26^ and in turn, better capture age-related differences in functional connectivity.

To facilitate comparisons between different MRI modalities, an innovative approach is to assess both structural and functional connectivity using graph theory,^27^ which divides the brain into networks comprised of anatomical nodes (i.e. GM regions) and the edges among them (i.e. WM connections). Graph theory has been a successful tool for identifying age-related differences in structural and functional connectivity, and for assessing relations between these measures.^17,18,20,28^ To date, however, no study has tested whether high-resolution structural connectivity better explains age-related variance in functional connectivity, when directly compared to standard resolution DWI.

Here, we used a sample of 140 healthy adults (18–88 years of age) who underwent resting-state fMRI (rsfMRI) and standard resolution DWI (1.5 mm^3^ voxels; 3.375 µl volume) and high-resolution DWI (1 mm^3^; 1 µl volume) data acquisition. An extensive test battery assessed both general and specific forms of fluid cognition, and graph theoretical analyses assessed structural and functional connectivity among cortical and sub-cortical networks.^29,30^ The goals of this study were 3-fold. First, we aimed to replicate our previous work using a subset of these data (*n* = 61) demonstrating that the 3-fold increase in resolution from the high-resolution relative to standard acquisition uniquely identified assessed patterns of age-related decreases in WM connectivity both within- and between-networks.^31^ We conducted these analyses across the entire brain to assess whether this pattern was more evident in certain networks, such as those with greater superficial WM regions (e.g. sensorimotor network) versus long-range connections (e.g. default mode network). Second, we aimed to replicate previous reports of age-related decreases in within-network functional connectivity, without any corresponding difference in between-network connectivity.^17,32^ We extended this work by testing whether these patterns are evident even when including adults aged 80+ years.^33^ Our third goal, representing a novel extension that has not yet been assessed in prior work,^31^ tested our primary hypothesis: that high-resolution structural connectivity should significantly mediate age-related differences in functional connectivity and fluid cognition, beyond standard resolution DWI.

Studies of healthy younger adults report stronger relations between structural and functional connectivity in primary sensory networks.^34^ However, studies of healthy aging report larger age-related differences in functional connectivity for association (e.g. default mode) than primary sensory (e.g. visual) networks.^32,35^ Thus, we expected that healthy aging may instead be associated with stronger structure-function relations for association than sensory networks,^36^ and that this may reflect their vulnerability to underlying age-related structural degradation.

## Materials and methods

### Participants

This study was conducted in compliance with the Code of Ethics of the World Medical Association (Declaration of Helsinki) for experiments involving humans and the protocol was reviewed and approved by the Institutional Review Board for Duke University Medical Center. All participants were compensated for their time and provided informed consent.

Participants were recruited from the Raleigh-Durham, North Carolina area and surrounding communities using a combination of printed or virtual flyers distributed to local hospitals, community centres and other local organizations. Study participation initially involved a screening and behavioural testing session, followed by an MRI scan that was acquired approximately one month later. Inclusion criteria were: at least a high school education, free of major medical conditions as assessed by a modified version of the Christensen *et al*.^37^ questionnaire (e.g. diabetes, uncontrolled hypertension, epilepsy, prior loss of consciousness and recent diagnosis of cancer), no current prescription medications known to affect cognition (e.g. stimulants and antidepressants), intact general cognition on the Mini-Mental State Exam^38^ (score ≥ 27; *n* = 61) or the Montreal Cognitive Assessment^39^ (score ≥ 26; *n* = 79), age-expected performance on the Wechsler Adult Intelligence Scale-III vocabulary subtest^40^ (score > 50th percentile), no evidence of major depression on the Beck Depression Inventory^41^ (score ≤ 16), and adequate visual acuity on the Freiburg Visual Acuity Test^42^ (Snellen score < 20/40).

For the initial screening session, 252 healthy adults from the community participated (18–88 years of age). We excluded 73 of these participants due to attrition (*n* = 8), failing to meet the inclusion criteria or MRI safety requirements (e.g. body size, claustrophobia, implanted ferrous metal; *n* = 63), or for being outliers (>3 standard deviations) on one or more of the cognitive tests (*n* = 2; described below, *Psychometric and Cognitive Tasks*). The remaining 179 participants completed MRI scanning, but we were prohibited from acquiring the high-resolution DWI data for 12 individuals due to time constraints. We excluded 27 additional participants due to extreme head motion, MRI artefacts or issues with image pre-processing, or poor performance (accuracy < 75%) on a visual attention task completed during fMRI scanning. Table 1 includes details on the final sample of 140 participants 18–88 years of age (51.4% female, 8.6% Hispanic, 83.6% White). The age distribution is as follows: 18–29 (*n* = 26), 30 s (*n* = 22), 40 s (*n* = 22), 50 s (*n* = 17), 60 s (*n* = 22), 70 s (*n* = 20) and 80 s (*n* = 11).

**Table 1:** Participant characteristics

Measure	Mean	(SD)	*r* with age	*P*-value
Education (years)	17.050	(2.249)	0.057	0.506
MMSE (*n* = 61)	29.541	(0.621)	0.004	0.978
MoCA (*n* = 79)	28.354	(1.197)	**−0.279**	**0.013**
BDI	3.514	(3.851)	0.030	0.723
Vocabulary	56.000	(4.686)	0.002	0.981
Colour vision	13.886	(0.368)	**−0.257**	**0.002**
Visual acuity	−0.015	(0.090)	**−0.367**	**<0.001**
Scan Interval	36.971	(27.444)	−0.036	0.675

The *n* = 140 for all tests except the Mini-Mental State Exam (MMSE)^38^ and Montreal Cognitive Assessment (MoCA).^39^ Values are presented as mean (SD). BDI = score on the Beck Depression Inventory^41^; Vocabulary = raw score on the vocabulary subtest of the Wechsler Adult Intelligence Scale III^40^; Colour Vision = score on Dvorine colour plates^43^; Visual Acuity = inversed logarithm of the minimum angle of resolution for the Freiburg visual acuity test,^42^ Scan Interval = number of days between the initial screening session and MRI scan. Significant effects are presented in bold.

### Psychometric and cognitive tasks

As described previously,^20,44-46^ participants completed a fluid cognition battery comprised of 12 individual tests. Perceptual-motor speed was measured by three computer-based reaction time (RT) tests (simple RT, two versions of choice RT), and number correct in 85 s. from the National Institutes of Health (NIH) Toolbox Pattern Comparison Test.^47^ Executive function was measured by the Digit Symbol Substitution Task, Flanker Task (incompatible versus compatible RT), Trail Making Test Part B versus A,^48^ and NIH Toolbox Card Sort Test.^47^ Memory was measured by a computer-based 6-item change detection task,^49^ 20-min. delayed verbal recall of 16 words, the Logical Memory Test,^40^ and the NIH Toolbox Picture Sequence Test.^47^

### Imaging data acquisition

Imaging data acquisition occurred at the brain imaging and analysis centre at Duke University Medical Center across two separate experiments of visual attention that occurred between 2021 and 2024. Most of the data (*n* = 92) were acquired on a 3T GE MR750 MRI scanner equipped with an eight-channel head coil. In 2022, the scanner underwent a series of upgrades, and the remainder of the data (*n* = 48) were acquired on a 3T GE Ultra High-Performance MRI scanner equipped with a 48-channel head coil. Participants wore earplugs to protect them from scanner noise, and head motion was minimized by using foam padding.

For 61 participants, a high-resolution T1-weighted image volume was acquired using a 3D fast inverse-recovery-prepared spoiled gradient recalled echo (SPGR) sequence with these parameters: voxel size = 0.5 × 0.5 × 0.5 mm, acquisition matrix = 512 × 512 × 292 mm, repetition time (TR) = 2200 ms, echo time (TE) = 3.1 ms, flip angle = 8°, sensitivity encoding (SENSE) factor = 2. For the remaining 79 participants, a high-resolution T1-weighted image volume was acquired using a 3D inverse-recovery-prepared SPGR sequence with the following parameters: TR = 2132.8 ms, TE = minimum full echo, flip angle = 8°, voxel size = 1 × 1 × 1 mm, acquisition matrix = 256 × 256 × 116 mm (*n* = 9) or 256 × 256 × 124 (*n* = 70) and SENSE factor = 2.

For all 140 participants, whole-brain standard resolution DWI data were acquired using a single-shot spin-echo echo planar imaging (EPI) sequence with these parameters: acquisition matrix = 144 × 144 × 83 mm, TR = 4620 ms, TE = 64.1 ms, flip angle = 90°, voxel size = 1.5 × 1.5 × 1.5 mm, SENSE factor = 1 and multi-band factor = 3. Diffusion-weighted gradients were applied in 90 directions with *b* values of 1500 s/mm^2^ and 3000 s/mm^2^ and with two non-diffusion-weighted *b* = 0 images. For most participants (*n* = 129), a second diffusion sequence was acquired with six phase-encoding directions of opposite polarity using the same parameters as the main sequence, except that TR = 4971 ms. Subsequent analyses of these data were limited only to the data acquired using the *b* = 1500 s/mm^2^ shell to facilitate more comparable analyses with the high-resolution data.

For all 140 participants, whole-brain high-resolution DWI data were acquired using a four-shot multiplexed sensitivity encoding (MUSE) sequence. For 74 participants, single-band data were acquired using a spin-echo EPI sequence with these parameters: voxel size = 1 × 1 × 1 mm, acquisition matrix = 256 × 256 × 90 mm (*n* = 61) or 256 × 256 × 116 mm (*n* = 13), TR = 13 000 ms, TE = 58 ms, flip angle = 90°, SENSE factor = 1 and multi-band factor = 1. Diffusion-weighted gradients were applied in 15 directions with a *b* value of 1000 s/mm^2^ (*n* = 61) or 800 s/mm^2^ (*n* = 13) and with two non-diffusion-weighted *b* = 0 s/mm^2^ images. For the remaining 66 participants, multi-band data were acquired using a spin-echo EPI sequence with these parameters: voxel size = 1 × 1 × 1 mm, acquisition matrix = 164 × 328 × 122 mm, TR = 8767 ms, TE = 60.8 ms, flip angle = 90°, SENSE factor = 1 and multi-band factor = 2. Diffusion-weighted gradients were applied in 25 directions with a *b* value of 800 s/mm^2^ and with two non-diffusion-weighted *b* = 0 images. For nearly all participants with multi-band data (*n* = 64), a second diffusion sequence of one *b* = 0 s/mm^2^ volume and one 800 s/mm^2^ volume was acquired with phase-encoding directions of opposite polarity using identical parameters.

For all 140 participants, rsfMRI data were acquired (eyes open, central fixation) using a T2*-weighted gradient-echo EPI sequence with these parameters: voxel size = 2 × 2 × 2 mm, acquisition matrix = 128 × 128 × 50 mm (*n* = 61) or 128 × 128 × 63 mm (*n* = 79), TR = 1500 ms, TE = 30 ms, flip angle = 60°, SENSE factor = 2 and number of volumes = 300. For the 79 participants with 63 slices acquired, a set of field maps were acquired using an EPI sequence with phase-encoding directions of opposite polarity and identical parameters to the rsfMRI data, except that TR = 1000 ms and flip angle = 90°.

During scanning, we also acquired data from task-related fMRI, susceptibility-weighted angiography, and fluid attenuated inversion recovery imaging, most of which has already been reported in separate articles.^17,45,46,50^

### Processing of DWI data

We first extracted the standard resolution DWI data limited only to the *b* = 1500 s/mm^2^ shell (*dwiextract*) to avoid the potential confounding effect of gradient strength in comparisons between the two acquisitions of interest.^51,52^ All DWI data were then pre-processed using MRtrix3^53^ based on the following sequence of steps: denoising (*dwidenoise*), correction for motion, eddy current-induced distortions and susceptibility-induced distortions (*dwifslpreproc*), bias-correction (*dwibiascorrect*) and generation of a skull-stripped whole-brain mask (*dwi2mask*). The only exception to this pipeline was that the correction of susceptibility-induced distortions was not performed for the single-band MUSE data because the conventional SENSE reconstruction procedure reconstructed full field-of-view images from four DWI segments and allowed for the calculation of shot-to-shot phase variations.^54^ A trained researcher visually inspected all processed DWI scans and deemed them to be acceptable for the coverage of the brain mask, sufficient correction of motion and susceptibility-induced distortions, the absence of MR artefacts (e.g. ghosting, radio frequency inhomogeneities), and no major anatomical abnormalities.

To estimate the structural connectome, we first used the Dhollander^55^ algorithm to estimate tissue-specific response functions from GM, WM and CSF (*dwi2response*). We then derived fibre orientation distribution (FOD) maps (*dwi2fod*) using multi-shell, multi-tissue constrained spherical deconvolution (*mstmt-csd*) based on two compartments (WM and CSF) because the data comprised only a single shell, and performed normalization (*mtnormalize)* of the resulting FOD maps.^53,56,57^ Next, we generated a GM/WM boundary mask based on the high-resolution T1-weighted image volume (*5ttgen* and *5tt2gmwmi*). For each DWI acquisition, the average skull-stripped, pre-processed *b*  *=* 0 s/mm^2^ image was registered to the GM/WM boundary mask using a six degrees of freedom linear registration in FSL’s *flirt.*^58-60^ The inverse of this transformation matrix was then used to register the GM/WM boundary mask to native diffusion space (*transformconvert*, *mrtransform*). Using the FOD maps, we generated 10 million tracts (*tckgen*) using anatomically constrained probabilistic tractography (minimum streamline length = 1 mm, maximimum length = 250 mm, FOD amplitude cutoff = 0.06). Using anatomically constrained probabilistic tractography helps ensures a biologically plausible termination of streamlines in seed regions across the brain (GM nodes within the Brainnetome atlas, described under Graph Theoretical Analyses) by forcing their termination within the GM/WM boundary mask.^61^ We used spherical-deconvolution informed filtering of tractograms 2 (*tcksift2*) to assign a weight to each streamline, relative to the underlying apparent fibre density.^62^ Instead of removing streamlines, this approach incorporates a weighted contribution of all streamlines to the spherical deconvolution diffusion model,^63^ represents the fibre density in each voxel more accurately, and helps prevent issues related to overfitting.^62^

### Processing of rsfMRI data

All rsfMRI data were pre-processed using fMRIPrep^64^ based on the following sequence of steps: generating a skull-stripped functional reference image from the first four image volumes, estimating susceptibility-induced distortions, estimating head-motion parameters with respect to the functional reference image (transformation matrices, and six corresponding rotation and translation parameters), and correcting for slice timing (AFNI’s *3dShift*). No spatial smoothing was performed.^65^ For the 61 participants that field maps were not acquired for, we used a boundary-based registration to correct for susceptibility-induced distortions by aligning the functional reference image to the high-resolution T1-weighted image volume with inverted intensity.^66^ For the remaining 79 participants, susceptibility-induced distortions were correcte4d by using *3dQwarp* to estimate the B0-non-uniformity map from the reverse polarity EPI field maps.^67^ Lastly, we resampled the corrected functional time-series onto the participants’ native space by applying a single, composite transform that was used for motion and susceptibility-induced distortion correction.

After processing in fMRIPrep, the rsfMRI data were then fed into xcpEngine (https://xcpengine.readthedocs.io/) to remove the first four brain volumes, perform confound regression, scrub high-motion volumes, and apply a temporal band-pass filter (0.01–0.08 Hz). Confound regression included frame-to-frame motion estimates, mean signals from WM and CSF, the mean global signal, and the temporal and quadratic derivatives of these signals, yielding 36 nuisance regressors.^68^ Before motion scrubbing, mean frame-wise displacement (FD)^69^ was 0.130 mm (SD = 0.053) and exhibited a positive correlation with age, *r* = 0.259, *P* = 0.002. We used spectral interpolation to scrub all resting-state volumes with FD > 0.50 mm. If fewer than five volumes remained between two outlier volumes, we scrubbed both outliers and all the intervening volumes to account for residual motion.^70-72^ Mean duration of the scrubbed rsfMRI data across all participants was 7.40 min; (SD = 0.24), with a minimum of 5.85 min.

### Graph theoretical analyses

To assess connectivity, we used a standard network partition based on rsfMRI data derived from a separate study of healthy younger adults.^29^ The networks were based on a parcellation of 210 cortical and 36 sub-cortical GM nodes from the Brainnetome atlas^30^ in Montreal Neurological Institute (MNI) 152 template space (1 mm^3^ resolution). For the estimation of both structural and functional connectivity, each cortical node was assigned one of the following seven networks: dorsal attention, default mode, frontoparietal, limbic, sensorimotor, ventral attention and visual networks.^29^ We combined the 36 sub-cortical nodes into a single sub-cortical network mask, similar to prior work.^46,73,74^ Unfortunately, the slice prescription for both the high-resolution DWI and the rsfMRI data from the first experiment (*n* = 61) did not have sufficient spatial coverage of inferior temporal regions, mostly in the limbic network, for ≥20 participants. In addition to its insufficient spatial coverage here, the limbic network is highly prone to EPI-related artefacts and high signal-to-noise ratios,^75^ and we therefore excluded the 26 limbic network nodes from our calculation of structural and functional connectivity, leaving a final set of 220 usable Brainnetome nodes. Applying the same functionally defined network partition for both the DWI and rsfMRI data allows for more direct comparisons between these modalities and aligns with our prior study that used this same partition to assess only structural connectivity.^31^ Our goal here was to assess how the measurement of structural connections between GM regions in various functional networks differed by DWI spatial resolution, rather than to characterize the unique modular structure of each imaging domain.

To assess structural connectivity for each participant and DWI acquisition type, the estimated streamlines in native diffusion space were transformed to standard MNI 152 1 mm^3^ space (*tcktransform*). We created symmetrical structural connectivity matrices by assigning the 10 million weighted streamlines to the final set of 220 Brainnetome nodes (*tck2connectome*), and then used a 2 mm radial search to asign the endpoint of each streamline to the nearest GM node.^62^ In the resulting unthresholded 220 × 220 matrices,^76^ the value in each cell corresponding to the sum of the weighted contribution of the streamlines connecting that pair of nodes (scaled by node volume).^77^

To assess resting-state functional connectivity for each participant, we used aligned the residualized image from xcpEngine (i.e. after motion scrubbing) to standard MNI 152 1 mm^3^ space using the *antsRegistration* tool. Symmetrical functional connectivity matrices were created using the correlations (Pearson *r*) of the time-series data for each of the 220 Brainnetome nodes, separately for each participant. In the resulting unweighted 220 × 220 matrices, negative correlations were set to zero,^27^ the diagonal was excluded, and the *r* values were transformed to Fisher *z* values.

Lastly, for the estimation of both structural and functional connectivity, we used the Brain Connectivity Toolbox^27^ to estimate graph theoretical measures of within-network connectivity (i.e. average connectivity strength among nodes belonging to a single network) and between-network connectivity (i.e. average connectivity strength between nodes belonging to a single network and all nodes beyond that network), separately for each of the seven networks of interest (i.e. excluding the limbic network).

### Experimental design and statistical analysis

Statistical analyses were primarily conducted within R Studio (version 2024.04.2) with a handful of analyses (i.e. partial correlations with more than 1 covariate) conducted within JASP (version 0.17.1). A *χ*^2^ test indicated that distribution of the different scanner features did not significantly differ among younger (18–39 years of age), middle-age (40–59 years of age) and older (60–88 years of age) adults, *χ*^2^ = 0.883, *P*_uncorrected_ = 0.643. However, scanner setup was nonetheless used as a covariate for all subsequent analyses with the MRI data. All results were corrected for multiple comparisons by applying false discovery rate (FDR) procedures.^78^

For the psychometric and cognitive data, the unrotated first factor derived from a principal axis factor analysis of all 12 tests was used to represent general fluid cognition, and memory, executive function, and perceptual-motor speed were represented by the first factor from each set of four tests within those individual domains.^9,20,79-81^ Our primary interest was the association between age and general fluid cognition and was tested using Pearson correlational analyses, but we also conducted similar exploratory analyses between age and each of the individual cognitive measures.

For the structural connectivity data, we first assessed the degree of similarity between the standard resolution and high-resolution measures for within- and between-network connectivity of each of the seven networks by conducting partial correlational analyses, while covarying for scanner setup. We then conducted partial correlational analyses between age and each of the seven network connectivity measures, separately for the standard resolution and high-resolution data, while covarying for scanner setup. We compared the size of the age-related correlations between the standard resolution and high-resolution acquisitions using the two-tailed Steiger’s *z* test approach for comparing correlated correlation coefficients.^82,83^

For the functional connectivity data, we conducted partial correlational analyses between age and each of the seven within- and between-network network connectivity measures, while covarying for scanner setup and head motion (FD). To test the hypothesis that high-resolution structural connectivity explains age-related differences in functional connectivity, beyond the standard resolution structural connectivity measures, we conducted parallel mediation analyses using the PROCESS macro in R Studio.^84^ Significant indirect effects, corresponding to the interaction between model paths, were determined by bias-corrected 95% confidence intervals (CIs) that did not contain zero after 10 000 bootstrap replacements. In these models, we included age as the predictor, scanner setup and head motion as covariates, functional connectivity as the outcome, and high-resolution and standard resolution structural connectivity measures as parallel mediators, with separate analyses for each network (e.g. frontoparietal structural connectivity as a mediator of age-related differences in frontoparietal functional connectivity). These parallel mediation analyses were conducted separately for measures of within- or between-network connectivity and were limited only to functional connectivity outcome measures that exhibited a significant association with age.

Lastly, we sought to understand whether any of the three forms of connectivity (resting-state functional, high-resolution structural and standard resolution structural) explained age-related differences in general fluid cognition. We again conducted parallel mediation analyses in the exact same way as above, except that now all three forms of connectivity were included as parallel mediators and general fluid cognition performance was modelled as the outcome variable. These models were again conducted separately for measures of within- or between-network connectivity, and the choice of mediator variables was limited only to measures of connectivity from networks that exhibited a significant association with age.

## Results

### Age-related differences in cognitive performance

**Figure 1:**
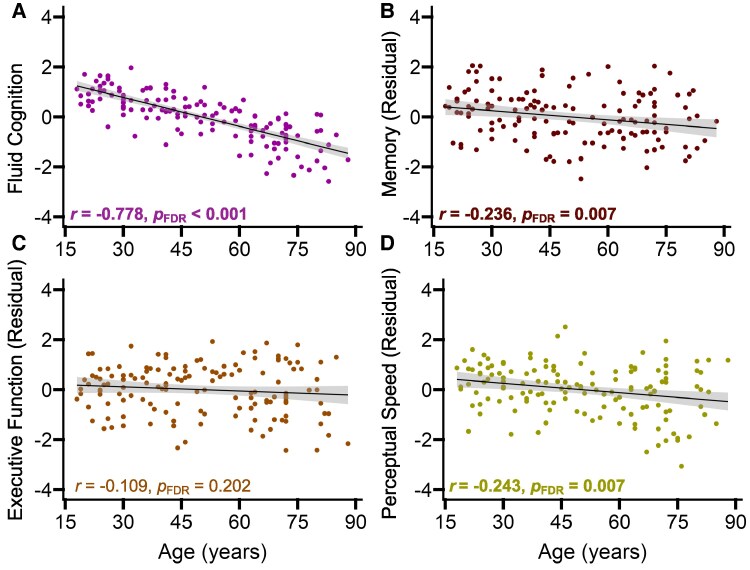
**Age-related differences in cognitive performance.** General fluid cognition was measured using a factor score from 12 separate tests of memory, executive function and perceptual-motor speed (*n* = 140). Memory, executive function and perceptual-motor speed were each measured using a factor score from the four domain specific subtests and then residualized for performance on the other two domains. Following false discovery rate (FDR) correction, Pearson correlations indicated significant age-related differences were evident for general fluid cognition (**A**), memory (**B**), and perceptual-motor speed (**D**), but not executive function (**C**). The shaded grey areas around the regression lines represent 95% confidence intervals. Each data point corresponds to an individual participant’s factor score for that particular cognitive domain.

To assess age-related differences in cognition, we focused on the general fluid cognition score but also explored the individual domain factor scores for memory, executive function and perceptual-motor speed. The individual domain factors were each significantly correlated with one another, Pearson *r* ≥ 0.479, *P*_FDR_ < 0.001. To account for unique domain-specific variance in performance, we residualized the individual domain scores to account for performance on the two factor scores outside of that domain.^20,79,81^ We then conducted Pearson correlations between age and each cognitive factor, with FDR correction applied for comparisons across four measures. Results indicated that increased age was significantly correlated with lower performance for general fluid cognition, perceptual-motor speed, and memory, but was not significantly related to executive function (Fig. 1).

### Structural connectivity

#### Correlations Between DWI Acquisitions

To examine associations between high-resolution and standard resolution structural connectivity, we used separate partial correlations (covaried for scanner setup), with FDR correction applied for comparisons across seven networks. Results indicated that the two acquisitions were generally highly correlated for within-network connectivity, with correlation coefficients ranging from 0.269 to 0.668 (Table 2; *r* High-Res—Standard). The correlations for between-network connectivity were also highly correlated, with correlation coefficients ranging from 0.375 to 0.743 (Table 2, *r* High-Res—Standard).

**Table 2:** Age correlations with white matter structural connectivity

	*r* Standard—age		*r* High-res—age		*r* High-res—standard		Steiger’s *z*	
Within-network connectivity								
VIS	**−0.405**	***	**−0.277**	**	**0.580**	***	1.771	
SMN	−0.178		**−0.468**	***	**0.463**	***	**3.589**	*
DAN	**−0.256**	**	−0.152		**0.668**	***	1.535	
VAN	−0.095		**−0.300**	***	**0.540**	***	**2.586**	*
FPN	0.122		−0.024		**0.463**	***	1.658	
DMN	**−0.216**	*	**−0.348**	***	**0.638**	***	1.916	
SUB	**−0.215**	*	**−0.447**	***	**0.269**	**	**2.465**	*
Between-network connectivity								
VIS	**−0.474**	***	**−0.500**	***	**0.743**	***	0.496	
SMN	**−0.312**	***	**−0.547**	***	**0.375**	***	**2.872**	*
DAN	**−0.351**	***	**−0.453**	***	**0.593**	***	1.479	
VAN	**−0.355**	***	**−0.519**	***	**0.494**	***	2.207	
FPN	**−0.416**	***	**−0.519**	***	**0.507**	***	1.429	
DMN	**−0.496**	***	**−0.584**	***	**0.607**	***	1.445	
SUB	**−0.550**	***	**−0.614**	***	**0.722**	***	1.286	

Networks are abbreviated as visual (VIS), sensorimotor (SMN), dorsal attention (DAN), ventral attention (VAN), frontoparietal (FPN), default mode (DMN), and sub-cortical (SUB). Asterisks correspond to the false discovery rate corrected *P*-value for the correlation coefficient presented in the preceding column. All correlations were covaried for scanner setup. Significant effects are presented in bold. *** = *P*_FDR_ < 0.001, ** = *P*_FDR_ < 0.01, * = *P*_FDR_ < 0.05.

To test whether these similarities were affected by age, we repeated the correlations using age as an additional covariate. The correlations between acquisitions generally remained the same for within-network connectivity, with correlation coefficients ranging from 0.197 to 0.659 (Supplementary Table 1). For between-network connectivity, the correlations generally decreased after included age as a covariate, but nonetheless remained strongly correlated, with correlation coefficients ranging from 0.257 to 0.663 (Supplementary Table 1).

#### Age-related differences

**Figure 2:**
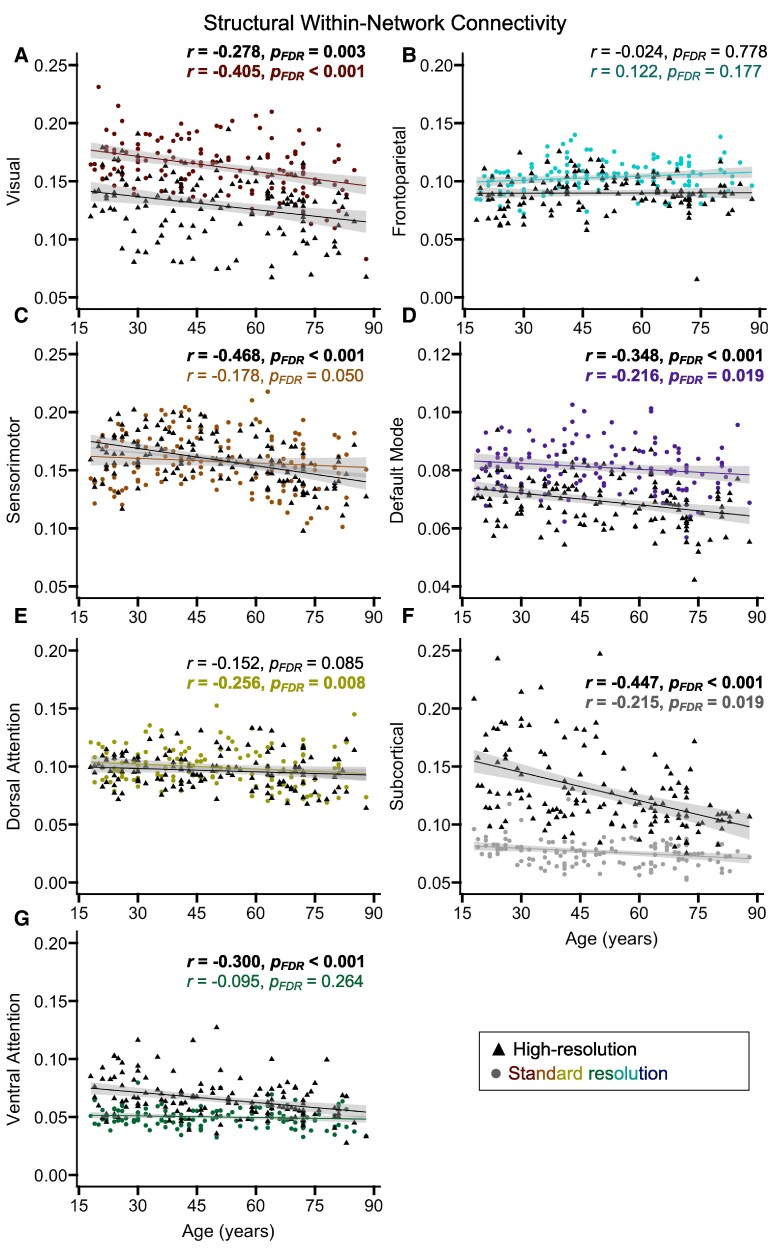
**Age-related differences in within-network structural connectivity.** Scatterplots display associations between age and within-network connectivity for each network, shown in Panels (**A**-**G**), separately for the high-resolution (black triangles) and standard resolution (coloured circles) data (*n* = 140). The shaded grey areas around the regression lines represent 95% confidence intervals. Significant effects from partial correlation analyses are presented in bold. The reported correlation coefficients were covaried for the type of scanner setup used. Each data point corresponds to an individual participant’s measure of structural connectivity for that particular network. FDR = false discovery rate.

**Figure 3:**
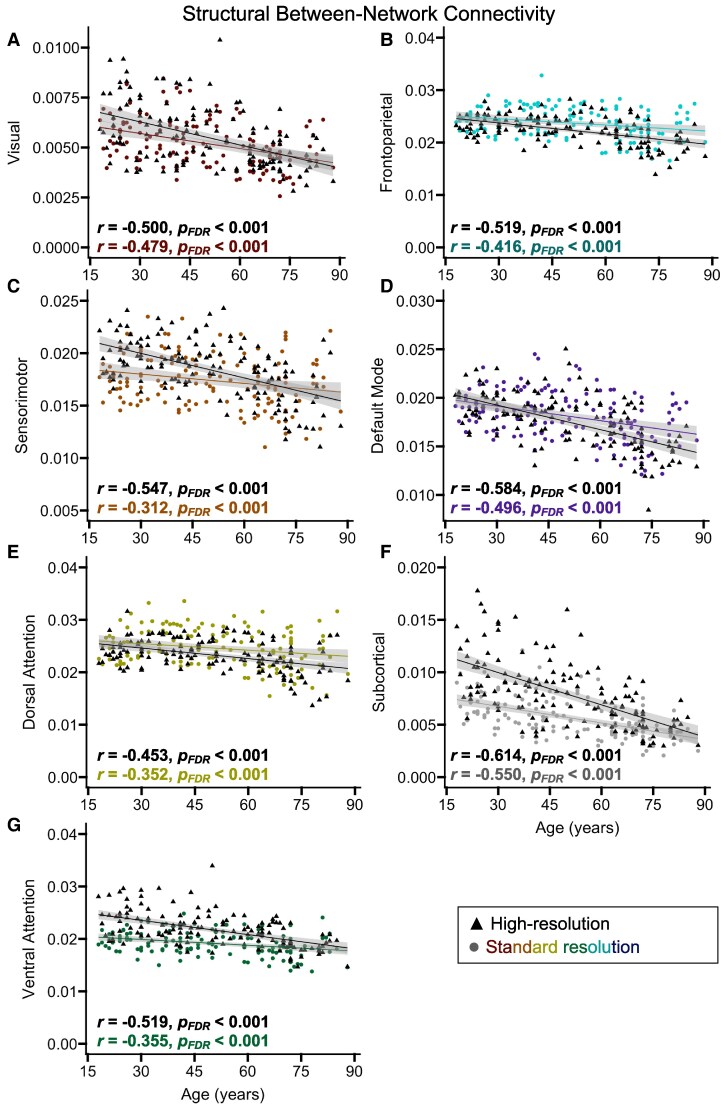
**Age-related differences in between-network structural connectivity.** Scatterplots display associations between age and between-network connectivity for each network, shown in Panels (**A**-**G**), separately for the high-resolution (black triangles) and standard resolution (coloured circles) data (*n* = 140). The shaded grey areas around the regression lines represent 95% confidence intervals. Significant effects from partial correlation analyses are presented in bold. The reported correlation coefficients were covaried for the type of scanner setup used. Each data point corresponds to an individual participant’s measure of structural connectivity for that particular network. FDR = false discovery rate.

To assess age-related differences in structural connectivity, we conducted partial correlations between age and within- or between-network connectivity, separately for each acquisition type, while covarying for scanner setup and applying FDR correction for comparisons across seven networks. For within-network connectivity (Fig. 2), the high-resolution data yielded significant age-related decreases for all networks except the dorsal attention and frontoparietal networks (Table 2; *r* High-Res—Age), and the standard resolution data yielded significant age-related decreases for all networks except the ventral attention and frontoparietal networks (Table 2; *r* Standard—Age). For between-network connectivity (Fig. 3), both the high-resolution (Table 2; *r* High-Res—Age) and standard resolution (Table 2; *r* Standard—Age) data yielded significant age-related decreases for all networks.

To determine whether the magnitude of age-related decreases in structural connectivity significantly differed between the standard resolution and high-resolution acquisitions, we compared the correlated correlation coefficients using two-tailed Steiger’s *z* tests.^82,83^ These results indicated that the age-related correlations were significantly larger for the high-resolution relative to standard resolution data for within-network connectivity in the sensorimotor, ventral attention and sub-cortical networks, and between-network connectivity in the sensorimotor network (Table 2; Steiger’s *z*).

### Functional connectivity

**Figure 4:**
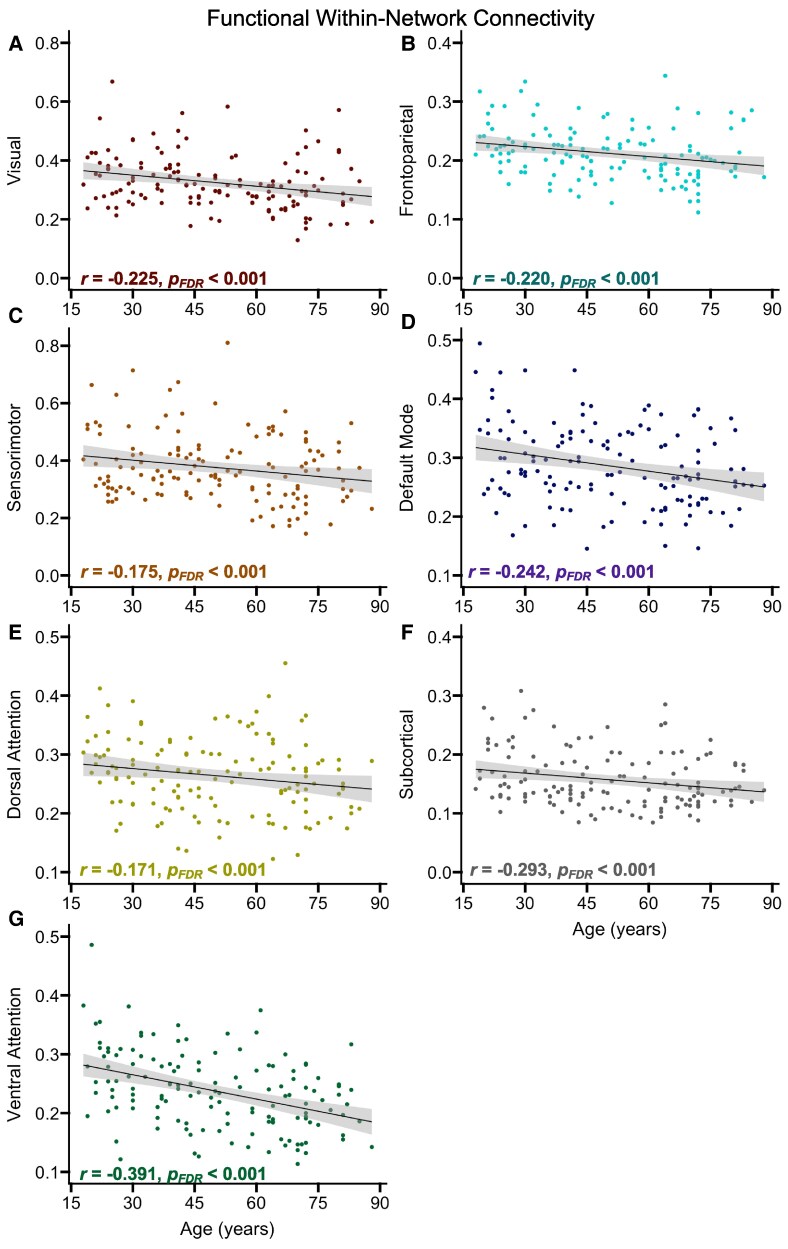
**Age-related differences in within-network functional connectivity.** Scatterplots display associations between age and within-network connectivity for each network, shown in Panels (**A**-**G**) (*n* = 140). The shaded grey areas around the regression lines represent 95% confidence intervals. Significant effects from partial correlation analyses are presented in bold. The reported correlation coefficients were covaried for the type of scanner setup used as well as head motion (FD). Each data point corresponds to an individual participant’s measure of functional connectivity for that particular network. FDR = false discovery rate.

To assess age-related differences in functional connectivity, we conducted separate partial correlations between age and within- or between-network connectivity, while covarying for scanner setup and head motion and applying FDR correction for comparisons across seven networks. For within-network connectivity, results indicated that increased age was significantly associated with lower connectivity in each of the individual networks (Fig. 4). However, there were no significant associations between age and between-network connectivity for any of the individual networks, *r* ≤ 0.120, *P*_FDR_ ≥ 0.562.

Based on prior reports of larger age-related differences in graph theoretical measures of functional connectivity for association relative to primary sensory networks,^32,35^ we created an average measure of within-network connectivity for association networks (frontoparietal, default mode, dorsal attention and ventral attention) and compared age-related differences in this measure to age-related differences within-network connectivity for the visual and sensorimotor networks. We used the same two-tailed Steiger’s *z* approach as above and applied FDR correction for two comparisons. Results indicated that the age-related difference was significantly larger for the association networks relative to the sensorimotor network, *z* = 2.226, *P*_FDR_ = 0.046, but was not significantly different from the age-related difference in the visual network, *z* = 1.664, *P*_FDR_ = 0.096.

### Structural versus functional connectivity

Next, we sought to test whether structural connectivity mediated age-related differences in functional connectivity. We limited these analyses to measures of within-network connectivity that were significantly related to age for the functional, high-resolution structural and standard resolution structural data (i.e. visual, default mode, and sub-cortical). For each of the three networks, we constructed mediation models with age as the predictor variable, within-network functional connectivity as the outcome variable, and the high-resolution within-network structural connectivity measure and the corresponding standard resolution version of it as parallel mediators. This parallel approach provides a direct comparison of the mediation effects for the measures derived from the two DWI acquisitions because the measures are covaried for each other.

Results indicated that high-resolution structural connectivity within the default mode network was a significant mediator of age-related decreases in functional connectivity within the default mode network (Table 3 and Supplementary Fig. 1). The beta coefficient for the indirect effect was negative (β = −0.0003), reflecting the product of one negative and one positive effect: the decrease in functional connectivity with increasing age (the *a* path), and the increase in within-network functional connectivity with increasing within-network structural connectivity (the *b* path). Thus, age influenced the decrease in default mode functional connectivity indirectly, through the decrease in structural connectivity, which was detectable with the higher resolution MUSE sequence.

**Table 3:** Mediation of default mode functional connectivity by structural connectivity

	Effect	SE	*t*	*P*	Lower CI	Upper CI
Age effect (a path)						
High-resolution	**−0.0001**	**0.00003**	**−4.041**	**< 0.001**	**−0.00021**	**−0.00007**
Standard resolution	**−0.0001**	**0.00004**	**−2.921**	**0.004**	**−0.00017**	**−0.00003**
Mediator to outcome (b path)						
High-resolution	**2.173**	**0.945**	**2.300**	**0.023**	**0.305**	**4.042**
Standard resolution	−1.836	0.938	−1.957	0.052	−3.692	0.020
Total effect for age (c path)						
	**−0.0009**	**0.0003**	**−2.904**	**0.004**	**−0.0015**	**−0.0002**
Direct effect for age (*c*′ path)						
	**−0.0008**	**0.0003**	**−2.407**	**0.017**	**−0.0014**	**−0.0001**
Mediation effect (*a* × *b* path interaction)						
Total	−0.0001	0.00012	**−**	**−**	−0.00037	0.00010
High-resolution	**−0.0003**	**0.00016**	**−**	**−**	**−0.00064**	**−0.00004**
Standard resolution	0.0002	0.00011	−	−	−0.000001	0.00044

Parallel mediation models with age as the predictor variable (*x*), functional connectivity within the default mode network as the outcome variable (*y*), and high-resolution and standard resolution structural connectivity within the default mode network as parallel mediators (*m*); *a* = predictor to mediator pathway; *b* = mediator to outcome pathway, while controlling for *a* path; *c* = total effect of predictor; *c’* = direct effect of predictor, controlling for mediators; *a x b* = interaction of *a* and *b* paths representing indirect influence of *x* on *y* as mediated by *m*; effect = unstandardized beta coefficient; Lower/Upper CI = lower/upper bounds of bias-corrected 95% confidence intervals. Significant effects are presented in bold.

### Connectivity versus cognition

Finally, we tested whether the connectivity measures mediated age-related differences in cognitive performance, specifically focusing on measures that were significantly correlated with age. For between-network connectivity, these were structural connectivity for each of the seven individual networks. For within connectivity, the relevant measures were structural connectivity from all networks except for the frontoparietal network, and functional connectivity from all seven networks. Similarly, we focused on the general fluid cognition factor as the outcome measure as it exhibited the largest correlation with age.

For between-network connectivity, we constructed separate mediation models for each of the seven networks with general fluid cognition performance as the outcome variable, high-resolution and standard resolution structural connectivity as parallel mediators, and scanner setup as a covariate of no interest. Results indicated that that the individual indirect effects for the high-resolution and standard resolution connectivity measures were not significant for any network (Table 4 and Supplementary Figs 2–4). However, the total indirect effect was significant for the models using structural connectivity from the sensorimotor, dorsal attention, or frontoparietal networks as mediators, indicating that the combined effect of the standard resolution and high-resolution connectivity measures significantly mediated age-related differences in general fluid cognition (Table 4). The individual or total indirect effects were not significant for the other four networks.

**Table 4:** Mediation of fluid cognition by between-network structural connectivity

	Effect	SE	*t*	*P*	Lower CI	Upper CI
Model 1: Sensorimotor between-network connectivity						
Age effect (a path)						
High-resolution	**−0.00008**	**0.00001**	**−7.647**	**< 0.001**	**−0.00010**	**−0.00006**
Standard resolution	**−0.00004**	**0.00001**	**− 3.844**	**< 0.001**	**−0.00006**	**−0.00002**
Mediator to outcome (b path)						
High-resolution	32.181	23.109	1.393	0.166	−13.522	77.884
Standard resolution	40.707	23.702	1.717	0.088	−6.169	87.582
Total effect for age (c path)						
	**−0.039**	**0.003**	**−14.325**	**< 0.001**	**−0.044**	**−0.033**
Direct effect for age (c’ path)						
	**−0.035**	**0.003**	**−10.810**	**< 0.001**	**−0.041**	**−0.028**
Mediation effect (a x b path interaction)						
Total	**−0.004**	**0.002**	**−**	**−**	**−0.0080**	**−0.0006**
High-resolution	−0.003	0.002	−	−	−0.0067	0.0011
Standard resolution	−0.002	0.001	−	−	−0.0036	0.0002
Model 2: Dorsal attention between-network connectivity						
Age effect (a path)						
High-resolution	**−0.00007**	**0.00001**	**−5.942**	**< 0.001**	**−0.00010**	**−0.00005**
Standard resolution	**−0.00006**	**0.00001**	**−4.399**	**< 0.001**	**−0.00008**	**−0.00003**
Mediator to outcome (b path)						
High-resolution	31.355	21.795	1.439	0.153	−11.749	74.459
Standard resolution	28.107	20.471	1.373	0.172	−12.377	68.592
Total effect for age (c path)						
	**−0.039**	**0.003**	**−14.325**	**< 0.001**	**−0.044**	**−0.033**
Direct effect for age (c’ path)						
	**−0.035**	**0.003**	**−11.680**	**< 0.001**	**−0.041**	**−0.029**
Mediation effect (a x b path interaction)						
Total	**−0.0039**	**0.0016**	−	−	**−0.0073**	**−0.0009**
High-resolution	−0.0023	0.0017	−	−	−0.0059	0.0007
Standard resolution	−0.0016	0.0012	−	–	−0.0041	0.0005
Model 3: Frontoparietal between-network connectivity						
Age effect (a path)						
High-resolution	**−0.00007**	**0.00001**	**−7.100**	**< 0.001**	**−0.00009**	**−0.00005**
Standard resolution	**−0.00005**	**0.00001**	**−5.348**	**< 0.001**	**−0.00007**	**−0.00003**
Mediator to outcome (b path)						
High-resolution	37.148	24.059	1.544	0.125	−10.434	84.729
Standard resolution	37.659	24.600	1.531	0.128	−10.992	86.309
Total effect for age (c path)						
	**−0.039**	**0.003**	**−14.325**	**< 0.001**	**−0.044**	**−0.033**
Direct effect for age (c’ path)						
	**−0.034**	**0.003**	**−10.747**	**< 0.001**	**−0.040**	**−0.028**
Mediation effect (a x b path interaction)						
Total	**−0.005**	**0.002**	**–**	**–**	**−0.0092**	**−0.0010**
High-resolution	−0.003	0.002	–	–	−0.0070	0.0007
Standard resolution	−0.002	0.001	–	–	−0.0048	0.0004

Mediation models with age as the predictor variable (x), fluid cognition as the outcome variable (y), and high-resolution and standard DWI between-connectivity measures as parallel mediators (m); a = predictor to mediator pathway; b = mediator to outcome pathway, while controlling for a path; c = total effect of predictor; c’ = direct effect of predictor, controlling for mediators; a x b = interaction of a and b paths representing indirect influence of x on y as mediated by m; effect = unstandardized beta coefficient; Lower/Upper CI = lower/upper bounds of bias-corrected 95% confidence intervals. Significant effects are presented in bold.

For within-network connectivity, we constructed similar mediation models with standard and high-resolution structural connectivity, plus resting-state functional connectivity, as parallel mediators, with resting-state motion as an additional covariate. Results indicated a significant individual indirect effect for high-resolution structural connectivity from the sensorimotor network, indicating that age-related decline in structural connectivity within the sensorimotor network, detected by the high-resolution acquisition, mediated age-related differences in fluid cognition (Table 5 and Supplementary Fig. 5). The total indirect effect from the sensorimotor model was also significant, reflecting the combined contribution of the structural and functional connectivity measures to age-related differences in fluid cognition. The total or individual indirect effects were not significant for the remaining models assessed.

**Table 5:** Mediation of fluid cognition by within-network sensorimotor connectivity

	Effect	SE	*t*	*P*	Lower CI	Upper CI
Age effect (*a* path)						
High-resolution	**−0.0005**	**0.0001**	**−6.000**	**< 0.001**	**−0.00068**	**−0.00034**
Standard resolution	**−0.0002**	**0.0001**	**−2.182**	**0.031**	**−0.00042**	**−0.00002**
Resting-state	**−0.0011**	**0.0005**	**−2.070**	**0.040**	**−0.00212**	**−0.00005**
Mediator to outcome (*b* path)						
High-resolution	**6.568**	**3.026**	**2.171**	**0.032**	**0.583**	**12.552**
Standard resolution	3.502	2.542	1.378	0.171	−1.525	8.530
Resting-state	−0.203	0.443	−0.457	0.648	−1.080	0.675
Total effect for age (*c* path)						
	**−0.038**	**0.003**	**−13.613**	**< 0.001**	**−0.044**	**−0.032**
Direct effect for age (*c*′ path)						
	**−0.034**	**0.003**	**−10.984**	**< 0.001**	**−0.040**	**−0.028**
Mediation effect (*a* × *b* path interaction)						
Total	**−0.0039**	**0.0017**	**–**	**–**	**−0.0077**	**−0.0009**
High-resolution	**−0.0034**	**0.0016**	**–**	**–**	**−0.0069**	**−0.0006**
Standard resolution	−0.0008	0.0008	–	–	−0.0028	0.0003
Resting-state	0.0002	0.0005	–	–	−0.0008	0.0013

Mediation models with age as the predictor variable (*x*), fluid cognition as the outcome variable (*y*), and high-resolution DWI, standard resolution DWI, and resting-state within-connectivity measures from the sensorimotor network as parallel mediators (*m*); *a* = predictor to mediator pathway; *b* = mediator to outcome pathway, while controlling for *a* path; *c* = total effect of predictor; *c*′ = direct effect of predictor, controlling for mediators; *a* × *b* = interaction of *a* and *b* paths representing indirect influence of *x* on *y* as mediated by *m*; effect = unstandardized beta coefficient; Lower/Upper CI = lower/upper bounds of bias-corrected 95% confidence intervals. Significant effects are presented in bold.

## Discussion

Previous studies report that higher spatial resolution DWI better estimates the structural connectome relative standard resolution DWI. We translated these findings to healthy neurocognitive aging across the adult lifespan using a high-resolution DWI protocol that achieved a 3-fold increase in spatial resolution relative to a standard DWI protocol. We found that high-resolution DWI identified larger age-related decreases in WM structural connectivity than would be detected using standard resolution DWI alone. High-resolution structural connectivity also helped better explain age-related decreases in functional connectivity and fluid cognition, when directly compared to standard resolution DWI. These findings illustrate the utility of this high-resolution MRI protocol for detecting age-related alterations in brain function and cognitive performance, which will help set the stage for future studies wishing to capitalize on this methodology for further translational aging research.

### Structural connectivity and healthy aging

Increasing adult age was significantly associated with decreased WM structural connectivity across cortical and sub-cortical networks when assessed by both DWI acquisitions. When assessed by both acquisitions, age-related decreases in between-network connectivity were significant for all networks, but the difference in within-network connectivity was only present for the visual, default mode, and sub-cortical networks. However, the high-resolution acquisition identified significantly larger age-related differences in connectivity within the sensorimotor, ventral attention and sub-cortical networks, and for sensorimotor between-network connectivity. Finding larger, more widespread age-related differences in between- than within-network structural connectivity replicates prior graph theoretical reports.^17,85,86^ Extending this work, our findings suggest that age-related differences in structural connectivity may be better detected by high-resolution relative to standard resolution DWI acquisitions, in line with studies of animal models and healthy younger adults.^24-26^

Connectivity measures between the two acquisitions were highly correlated, similar to our previous study using a subset of these data (*n* = 61) that did not assess relations to functional connectivity or age-related differences in structural connectivity into the ninth decade of life.^31^ Interestingly, our prior study observed larger age-related correlations for structural connectivity when assessed by standard relative to high-resolution DWI, but the current study instead found larger age-related correlations for a few networks when assessed by high-resolution DWI. This divergence could be attributable to several potential differences, including the larger sample size, the combination of single-band and multi-band high-resolution data (the latter of which also had a greater number of diffusion-weighted directions and therefore, higher angular resolution), and the inclusion of adults beyond 80 years of age.

### Structural connectivity and its relation to functional connectivity

Beyond structural connectivity, we also identified significant age-related differences in within-network functional connectivity for all networks, whereas between-network connectivity was not significantly related to age for any network. This finding replicates and extends prior work^17,32^ by demonstrating that this pattern persists even when including ‘oldest-old’ adults beyond 80 years of age, who may exhibit more magnified effects of age on brain function.^33^ Although some studies report age-related increases in between-network functional connectivity,^18,21,35,87,88^ our current finding from a high-functioning sample of community-dwelling volunteers instead supports recent evidence suggesting that the pattern of increased between-network connectivity may be more characteristic of pathological aging, such as Alzheimer’s disease,^89^ rather than healthy aging. Between-network connectivity, by definition, averages connectivity strength between nodes from one network and nodes outside of that network. As such, it is possible that age-related increases in connectivity between one pair of networks and age-related decreases connectivity between another pair of networks results in a net zero effect of age on between-network connectivity, as observed here. This possibility could be tested in future studies disaggregating the pairwise associations of connectivity between each combination of network. Some studies further report larger age-related effects on functional connectivity for association relative to sensory networks,^32,35,89^ a pattern that was replicated here for association networks relative to the sensorimotor network.

Theoretical accounts would propose that the larger age-related alterations in association network functional connectivity should relate to the underlying structural architecture of association networks,^12,13^ which also exhibit relatively greater degradation than primary sensory cortices in healthy aging.^90,91^ However, empirical studies report mixed results on the association between measures of brain structure and function in adults across the lifespan.^14-16^ We suspected that these mixed findings may partly reflect the lower spatial resolution of standard DWI acquisitions limiting the ability to assess connectivity in fine-grained WM regions.^92,93^ We found that age-related differences in functional connectivity within the DMN (an association network) were significantly mediated by structural connectivity within the DMN, but only when assessed by high-resolution DWI. This finding suggests that the contribution of structural degradation to age-related alterations in brain function may be better detected by high-resolution DWI, and that measurement error in standard DWI may attenuate the ability to detect significant associations between WM connectivity and fMRI measures of brain function. This finding extends our previous work in a subset of the current sample that was limited only to analyses of structural connectivity, and to our knowledge, represents the first direct comparison between two different DWI measures of structural connectivity and their relation to functional connectivity.

Interestingly, the DMN comprises widely distributed anterior (e.g. superior and inferior frontal cortex) and posterior (e.g. precuneus, inferior parietal lobule) sub-regions that are uniquely impacted by Alzheimer’s-disease neuropathologies (e.g. amyloid burden), even years before the manifestation of cognitive impairment.^94,95^ However, posterior sub-regions of this network are among the first to accumulate amyloid and have been linked to aberrant functional connectivity even in healthy adults.^96^ The network partition employed here combined anterior and posterior sub-divisions of the default mode network^29^ and biomarker measures of neuropathologies were not collected from the current sample. Although the current sample was well-characterized with respect to general cognitive function (e.g. more than 12 psychometric tests in addition to standard tests of general cognition and vocabulary), it is important for future work to repeat these analyses in a sample with measures of biomarker status (e.g. amyloid and tau positivity) so that the effect of neuropathologies on functional and structural connectivity can be directly assessed, especially within the distinct anatomical sub-divisions of the DMN.

Previous studies have further demonstrated the utility of graph theory for assessing relations between structural and functional connectivity.^17,18,20,28^ Our current results are in line with prior suggestions that structural and functional connectivity may be relatively independent,^17,20^ and that these relations may specifically be network dependent. Studies of healthy younger adults report stronger relations between structural and functional connectivity in sensory than association networks,^34^ but the current finding is in line with prior studies reporting stronger relations for association than sensory networks in healthy aging.^36,97^ Together, these findings suggest that DMN functional connectivity is related to underlying age-related degradations in structural connectivity, which may be better detected by high-resolution DWI.

### Structural connectivity, functional connectivity, and fluid cognition

The age-related decrease in structural connectivity mediated age-related decline in general fluid cognition, a measure that exhibited a strong negative relation with age (with some degree of unique decline in memory and perceptual-motor speed). Parallel mediation analyses indicated that the combination of high-resolution and standard resolution DWI measures of between-network connectivity from sensorimotor, dorsal attention and frontoparietal networks helped explain age-related decreases in fluid cognition, together supporting the notion of cortical disconnection as a mechanism underlying normal age-related cognitive decline.^6,8-10^ Importantly, high-resolution structural connectivity within the sensorimotor network was a significant individual mediator of age-related differences in fluid cognition, and this network also exhibited a significantly larger age-related correlation between assessed by high-resolution than standard DWI. However, a significant total effect for this sensorimotor model indicated that standard resolution and functional within-network connectivity also exerted some degree of combined influence on age-related differences in cognitive performance. Together, these findings suggest that WM plays a crucial role in fluid cognition across the adult lifespan, and that high-resolution DWI may better detect this relation, at least for measures of within-network connectivity that likely comprise smaller, more fine-grained fibres.^93^

### Limitations and future research

This study is strengthened by the inclusion of adults across the lifespan, including adults beyond 80 years of age who are underrepresented in MRI studies of brain and cognitive aging. The high-resolution DWI acquisitions used here hold promise for bridging clinical and translational research as it requires a substantially shorter scan time than most high-resolution protocols but can nonetheless be acquired from animal models. However, comparisons between the standard and high-resolution data here may be confounded by the higher *b*-value in the standard data may better capture hindered and restricted sources of diffusion,^51,52^ which is why we excluded standard resolution data from the *b* = 3000 s/mm^2^ shell. Our analyses are also limited by the change in scanner setup that occurred during data collection. Although we included scanner setup as a covariate of no interest, we cannot fully rule out its impact on the current results. This change is scanner setup was also highly confounded with the upgrade from the single-band to multi-band high-resolution DWI data acquisition. Ninety-two percent of participants with single-band data were also scanned using the original scanner setup and 64% of participants with multi-band data were scanned using the upgraded scanner setup. By including scanner setup as a covariate of no interest in all analyses, this feature of the study also provides some confidence that the improved ability of high-resolution DWI to explain age-related differences in functional connectivity and fluid cognition cannot be solely attributed to differences between the high-resolution protocols. This feature does, however, limit our ability to disentangle potential differences between the two protocols here, representing an important direction for future studies in this line of work. Lastly, the present analyses were limited to observations from a single point in time and must be replicated in future work employing longitudinal designs to ensure that these patterns reflect true age-related effects instead of factors such as cohort effects.^98,99^

## Conclusions

Higher resolution MRI protocols may better capture the complex intricacies of neuroanatomy *in vivo*, but often at the expense of extensive scanning time. Here, we demonstrate the utility of a clinically feasible high-resolution DWI acquisition for understanding interactions among brain structure, brain function, and cognition in healthy aging. Compared to standard resolution DWI, we found that high-resolution structural connectivity better explained age-related variance in functional connectivity within the DMN and improved the ability to explain age-related variance in fluid cognition. Our results should inform future intervention work aimed at identifying adults who are at heightened risk for cognitive decline and should be informative for future studies that may wish to use this high-resolution DWI protocol in both human and animal models of neurocognitive aging.

## Supplementary Material

fcaf376_Supplementary_Data

## Data Availability

Because the community-dwelling adult participants did not consent to sharing their data, we are unable to make the raw data presented in this paper freely available online. The code that was used for data analyses is available on the Open Science Framework at https://osf.io/ck7nd/.
