# Supplementary material for: High-resolution structural connectivity mediates age-related differences in functional connectivity and fluid cognition
Source: Brain Commun. 2025 Sep 28;7(5):fcaf376. doi: 10.1093/braincomms/fcaf376 (PMC12528985; doi:10.1093/braincomms/fcaf376)
Supplement: fcaf376_Supplementary_Data [file fcaf376_supplementary_data.docx]

**Supplementary Figure 1**. Visual representation of mediation model. A graphical illustration is provided for the significant model reported in Table 3 of the main text. Solid or dashed lines indicate significant or non-significant effects, respectively. All effects are reported as the unstandardized beta coefficient *b* ± standard error along with the corresponding **p**-value. *a* = path from predictor to mediator; *b* = path from mediator to outcome, controlling for *a* path; *c* = total effect of predictor; *c’* = direct effect of predictor, controlling for mediators.

**Supplementary Figure 2**. Visual representation of mediation model. A graphical illustration is provided for the significant sensorimotor model reported in Table 4 of the main text. Solid or dashed lines indicate significant or non-significant effects, respectively. All effects are reported as the unstandardized beta coefficient *b* ± standard error along with the corresponding **p**-value. *a* = path from predictor to mediator; *b* = path from mediator to outcome, controlling for *a* path; *c* = total effect of predictor; *c’* = direct effect of predictor, controlling for mediators.

**Supplementary Figure 3**. Visual representation of mediation model. A graphical illustration is provided for the significant frontoparietal model reported in Table 4 of the main text. Solid or dashed lines indicate significant or non-significant effects, respectively. All effects are reported as the unstandardized beta coefficient *b* ± standard error along with the corresponding **p**-value. *a* = path from predictor to mediator; *b* = path from mediator to outcome, controlling for *a* path; *c* = total effect of predictor; *c’* = direct effect of predictor, controlling for mediators.

**Supplementary Figure 4**. Visual representation of mediation model. A graphical illustration is provided for the significant dorsal attention model reported in Table 4 of the main text. Solid or dashed lines indicate significant or non-significant effects, respectively. All effects are reported as the unstandardized beta coefficient *b* ± standard error along with the corresponding **p**-value. *a* = path from predictor to mediator; *b* = path from mediator to outcome, controlling for *a* path; *c* = total effect of predictor; *c’* = direct effect of predictor, controlling for mediators.

**Supplementary Figure 5**. Visual representation of mediation model. A graphical illustration is provided for the significant model reported in Table 5 of the main text. Solid or dashed lines indicate significant or non-significant effects, respectively. All effects are reported as the unstandardized beta coefficient *b* ± standard error along with the corresponding **p**-value. *a* = path from predictor to mediator; *b* = path from mediator to outcome, controlling for *a* path; *c* = total effect of predictor; *c’* = direct effect of predictor, controlling for mediators.

**Supplementary Table 1**

*Correlations between acquisitions covaried for age*

|  | *r* High-Res – Standard | | | |
| --- | --- | --- | --- | --- |
|  | *Within-network connectivity* | | *Between-network connectivity* | |
| VIS | **0.533** | *** | **0.663** | *** |
| SMN | **0.437** | *** | **0.257** | ** |
| DAN | **0.659** | *** | **0.519** | *** |
| VAN | **0.539** | *** | **0.387** | *** |
| FPN | **0.470** | *** | **0.375** | *** |
| DMN | **0.615** | *** | **0.450** | *** |
| SUB | **0.197** | * | **0.573** | *** |

*Note.* Networks are abbreviated as visual (VIS), sensorimotor (SMN), dorsal attention (DAN), ventral attention (VAN), frontoparietal (FPN), default mode (DMN), and subcortical (SUB). Asterisks correspond to the false discovery rate corrected *p*-value for the correlation coefficient presented in the preceding column. All correlations were covaried for scanner setup and age. Significant effects are presented in bold. *** = *p_FDR_* < 0.001, ** = *p_FDR_* < 0.01, * = *p_FDR_* < 0.05
